# Fairness-Aware Resource Allocation for Timely Information Dissemination in C-V2X Networks

**DOI:** 10.3390/s26206366

**Published:** 2026-10-09

**Authors:** Tongzhi Lin, Jiazhi Yang, Jinwei Zheng, Xianhao Shen

**Affiliations:** 1 College of Mathematics and Statistics, Guilin University of Technology, Guilin 541004, China; lintz@glut.edu.cn; 2 Guangxi Key Laboratory of Special Engineering Equipment and Control, Guilin 541004, China; jiazhi.yang@guat.edu.cn; 3 School of Mechanical and Electrical Engineering, Guilin University of Aerospace Technology, Guilin 541004, China; 4 School of Physics and Electronic Information Engineering, Guilin University of Technology, Guilin 541006, China; jinweizheng@glut.edu.cn

**Keywords:** resource management, Internet of Vehicles, multi-agent reinforcement learning, resource allocation

## Abstract

With the rapid development of intelligent transportation systems, the increasing number of connected vehicles and onboard sensing devices has generated substantial data traffic and placed stringent demands on communication resources in the Internet of Vehicles. In three-dimensional urban environments, physical blockage, high mobility, and co-channel interference can cause temporary link disruptions, preventing the timely dissemination of safety-critical vehicular information. This study investigates joint resource allocation for Cellular Vehicle-to-Everything (C-V2X) networks with heterogeneous vehicular platoons. We propose an Age of Information (AoI)- and Fairness-Aware Multi-Agent Resource Allocation (AF-MARL) 1 under imperfect and delayed channel state information. Its novelty lies in combining an AoI-dependent power cost with a cross-platoon AoI-dispersion penalty within a multi-task multi-agent reinforcement learning (MARL) architecture, thereby reducing unnecessary high-power transmission by favorable platoons while explicitly protecting disadvantaged platoons without adding Actor or Critic networks. Simulation results show that AF-MARL reduces interference and persistent AoI outages while maintaining reliable Cooperative Awareness Message (CAM) delivery under different platoon spacings and sizes.

## 1. Introduction

### 1.1. Background and Motivation

With the accelerating pace of global urbanization, issues such as traffic congestion [1], road accidents, and vehicle exhaust emissions have become increasingly severe, posing significant challenges to conventional road traffic management [2,3]. To address these challenges, countries worldwide are actively promoting the development of Intelligent Transportation Systems (ITS) [4]. As a core component of intelligent transportation, the Internet of Vehicles (IoV) [5] is experiencing unprecedented development opportunities, driven by the rapid advances in 5G/6G wireless communications, edge computing, and artificial intelligence.

With the increasing vehicle density and rapidly changing network topology [6], traditional resource allocation schemes based on centralized scheduling by base stations face substantial computational overhead and signaling delays. Consequently, distributed resource allocation based on Multi-Agent Reinforcement Learning (MARL) has emerged as an effective approach to addressing these challenges [7,8]. Existing studies have made significant progress in the joint optimization of Resource Block (RB) selection and power control by enabling multiple agents to continuously interact with the environment and learn through trial and error. However, most of these studies rely on idealized assumptions. In particular, they typically consider simplified two-dimensional (2D) homogeneous scenarios and assume that agents have access to perfect instantaneous Channel State Information (CSI). In realistic urban traffic environments, however, the physical space is inherently three-dimensional (3D) and highly heterogeneous. For example, the interleaved distribution of elevated roads and ground-level roads can result in substantially different physical blockage conditions for different vehicles. Moreover, the high mobility of vehicles introduces significant Doppler effects [9]. Combined with the inherent processing and communication delays of the system, these factors make it difficult for agents to obtain up-to-date CSI when making decisions, forcing them to rely primarily on historical channel state information.

When existing MARL algorithms are deployed in such realistic and challenging physical environments, their inherent limitations become increasingly apparent. In highly heterogeneous scenarios, conventional algorithms often lack effective fairness constraints. Consequently, vehicles on elevated roads with favorable Line-of-Sight (LoS) channels may transmit at high power to maintain their own transmission rates, thereby generating substantial co-channel interference. This interference can severely degrade the signals of disadvantaged ground vehicles operating under unfavorable Non-Line-of-Sight (NLoS) conditions, preventing timely data updates and causing their Age of Information (AoI) to exceed the prescribed threshold. Although some existing approaches attempt to minimize the average system-wide interference by introducing a Global Critic, such reward mechanisms may still allow the system to sacrifice the performance of a small number of disadvantaged vehicles as long as the overall interference metric is reduced. Such a trade-off is particularly problematic in autonomous driving scenarios, where stringent safety requirements demand reliable communication for all vehicles and leave little room for performance degradation of vulnerable users. Therefore, sacrificing the communication performance of disadvantaged vehicles to improve overall system-level metrics is potentially unacceptable and can pose critical risks to autonomous driving applications.

To overcome these challenges, this paper proposes the AoI- and Fairness-Aware Multi-Agent Resource Allocation (AF-MARL) algorithm for timely and reliable sensing-data dissemination in heterogeneous Cellular Vehicle-to-Everything (C-V2X) networks. The main contributions are threefold. First, we construct a simplified three-dimensional vehicular scenario that incorporates three-dimensional link distance, a fixed 10 dB excess-loss offset for ground-level Vehicle-to-Infrastructure (V2I) links, Doppler-correlated fast fading, and delayed CSI. Second, AF-MARL introduces an AoI-dependent power-cost mechanism at the agent level and an AoI-dispersion-based global fairness penalty at the system level. These complementary mechanisms encourage platoons with favorable channels to reduce unnecessary transmission power while preventing disadvantaged platoons from being sacrificed for improved average performance. Third, extensive simulations evaluate AoI freshness, Cooperative Awareness Message (CAM) transmission reliability, transmit-power distributions, and the interference floor under different intra-platoon spacings and platoon sizes, demonstrating the robustness and scalability of AF-MARL.

### 1.2. Related Work

With the rapid development of ITS, vehicular platooning [10,11] has gradually emerged as an important research topic. To satisfy the stringent requirements for highly reliable and low-latency communication in platooning scenarios, this problem has been extensively investigated in recent years. Wang et al. [12] addressed the resource allocation problem for multi-lane platooning and proposed a two-stage resource allocation strategy, together with a distributed dynamic programming-based algorithm for subchannel allocation and power control. Their approach aimed to maximize the number of supported platoons while minimizing system energy consumption. Peng et al. [13] employed evolved Multimedia Broadcast Multicast Service (eMBMS) and device-to-device (D2D) multicast communication to facilitate both intra-platoon and inter-platoon communications, thereby improving communication reliability and reducing latency in platooning scenarios. Efficient resource allocation for vehicular platoons also depends on the string stability of platoon motion. Zeng et al. [14] proposed a novel analytical framework for integrated communication and control systems. They derived the maximum tolerable communication delay for maintaining control-system stability and subsequently optimized the vehicle control parameters to ensure the reliability of the platooning system. Wang et al. [15] identified the potential threat posed by malicious lead vehicles to platoon stability and developed a two-stage platoon formation algorithm combined with a D2D resource allocation mechanism. By identifying malicious lead vehicles, their approach enabled the formation of stable platoons. Fernando et al. [16] proposed a fault-tolerant robust output-feedback control framework to mitigate platoon instability caused by practical factors such as vehicle heterogeneity and sensor measurement noise. The aforementioned studies mainly focus on inter- and intra-platoon communications or the stability of vehicular platoons. However, the highly dynamic nature of vehicular networks makes channel state estimation inherently dependent on historical and imperfect CSI. Meanwhile, the rapidly increasing number of connected vehicles makes it increasingly difficult for conventional optimization methods to efficiently handle the resulting high-dimensional and dynamically changing resource allocation problems. These challenges motivate the development of more adaptive and scalable approaches capable of effectively addressing complex vehicular communication environments.

Given the strong capability of Reinforcement Learning (RL) in complex decision-making and optimization under dynamic environments, its application to vehicular platooning systems has attracted considerable research attention. Reference [17] proposed an Actor-Critic-based RL approach and investigated the dynamic nature of vehicular environments and the continuous action space in IoV systems. Reference [18] developed a reinforcement learning method called HierNet to address resource allocation challenges in vehicular platoon networks. With the emergence of Deep Reinforcement Learning (DRL), its application to resource allocation has attracted increasing attention. Zhang et al. [19] developed a DRL-based resource allocation scheme to address the limited computing capabilities of onboard units and heterogeneous service requirements in autonomous driving scenarios. Xu et al. [20] proposed a multi-objective resource allocation framework based on DRL to improve the user experience of V2I links while ensuring reliable intra-platoon communications. Zhao et al. [21] combined DRL with Long Short-Term Memory (LSTM) networks to jointly optimize task offloading and resource allocation. References [22,23] incorporated multiple agents into the decision-making framework and modeled the policy search process using a Markov Decision Process (MDP). In a Multi-Agent System (MAS), multiple agents make autonomous decisions and coordinate with one another while operating in a shared environment. However, the conventional MDP formulation generally assumes that each agent updates its policy independently, making it insufficient to fully characterize the coupled decision-making process in MASs. In vehicular platooning systems, the timeliness of information is particularly critical, as obtaining essential information within a short period can directly affect traffic safety. In recent years, AoI has been increasingly adopted as a metric for characterizing information freshness in vehicular communication systems. References [24,25] proposed resource allocation schemes for vehicular communication at urban intersections and incorporated AoI as an important metric for evaluating their performance. Beyond terrestrial vehicular networks, Liu et al. [26] jointly optimized sensor transmission, node clustering, and UAV trajectory to minimize the long-term average AoI in UAV-assisted wireless-powered communication networks, demonstrating the close coupling among information freshness, communication scheduling, and mobility. At a broader interference-management level, Zanella et al. [27] showed that routing and power-control choices can substantially affect aggregate interference in ad hoc wireless networks. More recently, Sun et al. [28] proposed a state-aware V2X resource-allocation method that enriches the state representation and employs conditional attention to improve environmental perception and inter-agent cooperation. The above studies indicate that resource allocation in C-V2X networks is evolving from conventional optimization-based approaches toward multi-agent coordination. Meanwhile, AoI has emerged as a key metric for evaluating the effectiveness of resource allocation schemes. Nevertheless, most existing studies focus on evaluating resource allocation performance under relatively standardized or simplified scenarios. As real-world vehicular environments become increasingly complex and heterogeneous, whether these approaches can maintain robust performance under more realistic conditions remains an open challenge.

Recent AoI-aware MARL studies differ primarily in their optimization objectives and critic organizations. Reference [24] considers AoI-aware and energy-efficient C-V2X resource allocation, while [25] employs a cascaded multi-agent actor–critic design for AoI- and CAM-related objectives. Reference [29] introduces the M-MADDPG-TDEC framework, which combines centralized training with decentralized execution, twin global critics, task-specific local critics, TD3-based updates, and task decomposition for joint AoI and CAM resource allocation. Unlike the state-aware input-enhancement approach in [28], AF-MARL retains the architecture of [29] and focuses on reward-level modifications through an AoI-dependent power cost and a cross-platoon AoI-dispersion term. A systematic comparison of the architectures, objectives, observation and action spaces, and computational complexity is provided in Section 3.4.

## 2. System Model

This paper considers a vehicular platooning communication scenario at an urban intersection based on C-V2X technology. The system consists of a Roadside Unit (RSU) deployed at the center of the intersection and multiple independent vehicular platoons. Let the set of platoons in the system be denoted by, where each platoon consists of vehicles. The Platoon Leader (PL) is responsible for overall communication scheduling and management. The system bandwidth is divided into a set of orthogonal subchannels, denoted by. Regarding the communication mechanism, vehicles within each platoon frequently broadcast CAMs over Vehicle-to-Vehicle (V2V) links to maintain platoon stability. Meanwhile, the PL communicates with the RSU through a Vehicle-to-Infrastructure (V2I) link to periodically upload its status information and obtain global safety-related scheduling information.

**Figure 1:**
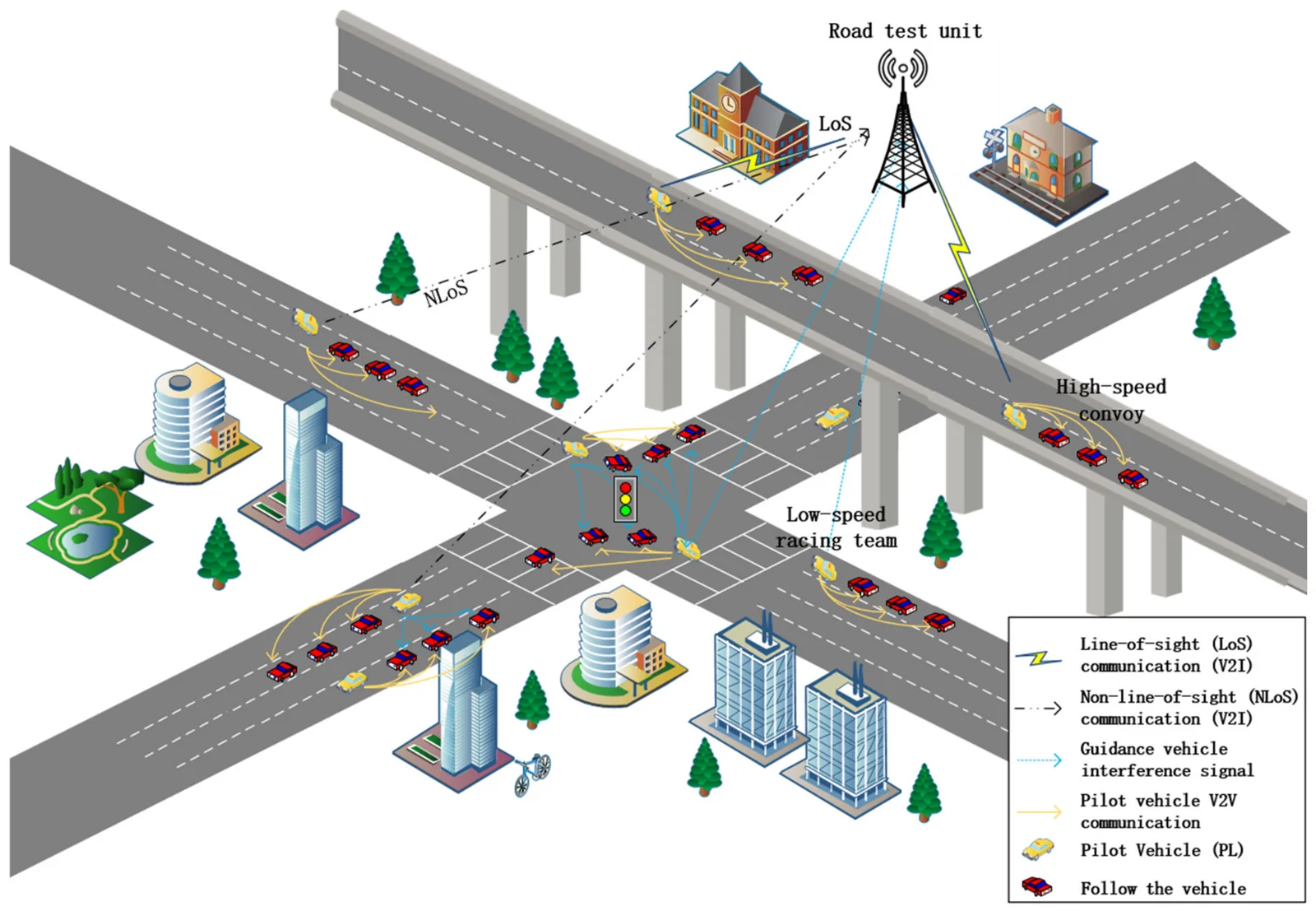
Three-dimensional heterogeneous C-V2X system model.

Unlike conventional two-dimensional scenarios, this paper constructs a simplified two-level three-dimensional topology to represent differences in road height and mobility between elevated-road and ground-level platoons, as illustrated in Figure 1. Along the vertical dimension, the vehicular platoons in the system are divided into two categories: elevated-road platoons and ground-level platoons. The elevated-road platoons travel on an overpass at a height of 10 m above the ground, whereas the ground-level platoons travel on conventional urban roads at a height of (m) m. In terms of mobility characteristics, these two types of platoons exhibit significant heterogeneity. The elevated-road platoons operate under relatively unobstructed, high-speed conditions, with their velocities ranging from 19 to 25 m/s while maintaining straight-line trajectories. In contrast, the ground-level platoons are constrained by complex urban traffic conditions, with velocities ranging from 4 to 10 m/s and a 40% probability of making a turn when passing through an intersection. This configuration provides a controlled heterogeneous scenario for evaluating resource allocation and is not intended as a geometry-resolved model of overpass and building blockage.

### 2.1. V2V and V2I Channel Models

To mitigate frequency-selective fading, the system employs Orthogonal Frequency Division Multiplexing (OFDM), where the total system bandwidth is divided into a set of orthogonal RBs, denoted by ϒ. In the time domain, the system is discretized into scheduling slots with a duration of Δ*t*. The channel gain on subchannel *k* for the PL of platoon *j* occupying the subchannel in time slot *t* can be expressed as

(1) $$h_{j}^{t}[k]=\alpha_{j}^{t}g_{j}^{t}[k]$$

where $\alpha_{j}^{t}$ denotes the large-scale fading, which consists of path loss and shadow fading, while $g_{j}^{t}[k]$ represents the small-scale fast fading.

Furthermore, the system follows the 3GPP C-V2X specifications (Release 14/15), with the carrier frequency *f_c_* set to 5.9 GHz. A frequency-dependent correction term 20log_10_(*f_c_*/2.0) is incorporated into the path loss model. Unlike conventional two-dimensional distance calculations, the proposed model employs the three-dimensional (3D) Euclidean distance $d_{3d}=\sqrt{d_{2d}^{2}+{\left(\Delta h\right)}^{2}}$ to characterize the physical distance between the transmitter and receiver. To represent a controlled difference between the two road levels, an additional fixed loss of 10 dB is applied to ground-level V2I links. This offset is a simplified average excess-loss assumption and does not vary with vehicle position, overpass geometry, building layout, or instantaneous LoS/NLoS conditions.

In highly mobile vehicular networks, the channel state exhibits strong temporal correlation. To avoid exposing the reinforcement learning agents to completely uncorrelated white noise, an AR(1) model [20] is adopted to characterize the temporal evolution of the fast-fading process. The severity of fast fading is directly determined by the Doppler shift *f_d_* = *v*/*λ* induced by the actual physical velocity *v* of the vehicle, where *λ* denotes the signal wavelength. Based on the Clarke reference model, the channel temporal correlation coefficient *ρ* over a time interval Δ*t* can be calculated using the zeroth-order Bessel function of the first kind as follows:

*ρ* = *J*_0_(2*πf_d_*Δ*t*) (2)

Based on this model, the temporal evolution of the small-scale fast-fading coefficient *g^t^* can be modeled as follows:

(3) $$g_{t}=\rho \cdot g^{t-1}+\sqrt{1-\rho^{2}}\cdot z^{t}$$

where *z^t^* denotes the additive Gaussian white noise. This model establishes an explicit relationship between physical vehicle mobility and channel fading dynamics. Since elevated-road and ground-level vehicles exhibit significant differences in their velocities, their corresponding Doppler shifts and temporal correlation coefficients *ρ* differ substantially. This further increases the complexity faced by the agents when observing and predicting the dynamics of the wireless environment. Together, these differences create persistent link asymmetry that exposes fairness failures hidden by average metrics and provides a controlled test of whether resource allocation can protect disadvantaged ground-level platoons.

### 2.2. Communication and Interference Model

At each scheduling slot *t*, the PL needs to make joint decisions regarding channel allocation and communication mode selection. Let the binary variable $\beta_{j,k}^{t}\in \{0,1\}$ indicate whether platoon *j* occupies subchannel *k* during time slot *t*. Meanwhile, a mode-selection variable $\theta_{j}^{t}\in \{0,1\}$ is introduced to indicate the communication mode of the PL. Specifically, when $\theta_{j}^{t}=0$, the PL communicates with the RSU through the V2I mode; when $\theta_{j}^{t}=1$, the PL broadcasts CAMs to the intra-platoon members through the V2V mode. According to Shannon’s capacity theorem, the instantaneous transmission rate of platoon *j* over the V2I link can be expressed as:

(4) $$C_{j,\mathrm{RSU}}^{t}[k]=\log_{2}(1+\frac{\left(1-\theta_{j}^{t})\beta_{j,k}^{t}p_{j}^{t}[k]h_{j,\mathrm{RSU}}^{t}[k\right]}{I_{j}^{t}[k]+\sigma^{2}})$$

where $p_{j}^{t}[k]$ denotes the transmit power, *σ*^2^ represents the background thermal noise power, and $I_{j}^{t}[k]$ denotes the total interference power within the system, which is given by:

(5) $$I_{j}^{t}[k]=\sum_{j^{*}}\beta_{j^{*},k}^{t}p_{j^{*}}^{t}[k]h_{j^{*},\mathrm{RSU}}^{t}[k]$$

where j^*^ ∈ P and *j* ≠ j^*^, the instantaneous transmission rate from the PL of platoon *j* to its *i* following vehicle over the V2V link is given by:

(6) $$C_{j,i}^{t}[k]=\log_{2}(1+\frac{\theta_{j}^{t}\beta_{j,k}^{t}p_{j}^{t}[k]h_{j,i}^{t}[k]}{I_{j,i}^{t}[k]+\sigma^{2}})$$

where $I_{j,i}^{t}[k]$ denotes the total interference power caused by external platoons, which is given by:

(7) $$I_{j,i}^{t}[k]=\sum_{j^{*}}\beta_{j^{*},k}^{t}p_{j^{*}}^{t}[k]h_{j^{*},i}^{t}[k]$$

where *j* ≠ j^*^ and *i* ∈ $N_{j}$. As described above, the 3D heterogeneous environment gives rise to a highly asymmetric interference relationship among communication links. Since elevated-road platoons enjoy favorable LoS channels, selfishly transmitting at high power to satisfy their own communication requirements can generate substantial interference $I_{j}^{t}[k]$ to other platoons. Meanwhile, ground-level platoons already suffer from a 10-dB penetration loss, resulting in a relatively weak desired signal at the receiver. When additional interference is introduced, the signal-to-interference-plus-noise ratio (SINR) of their V2I links can deteriorate sharply, potentially causing communication outages and preventing timely information updates.

### 2.3. Age of Information and Transmission Deadline Evolution Model

To comprehensively evaluate the communication reliability of the system, two evaluation models, namely AoI and a hard transmission deadline, are introduced according to the service characteristics of the V2I and V2V links, respectively. AoI is employed to quantify the freshness of the platoon state information maintained by the RSU. For platoon *j*, its AoI is denoted by $A_{j}^{t}$, representing the elapsed time since the last successful state update was received by the RSU. A state update is considered successful only when the transmission rate of the V2I link reaches or exceeds the minimum safety threshold *C*_min_ specified by the system. Accordingly, the discrete-time evolution of AoI is defined as follows:

(8) $$A_{j}^{t+1}=\left\{\begin{array}{cc} \Delta t, & \mathrm{if}\left(1-\theta_{j}^{t}\right)\beta_{j,k}^{t}C_{j,\mathrm{RSU}}^{t}[k]\geq C_{\min}\\ \min(A_{j}^{t}+\Delta t,A_{\max}), & \mathrm{other} \end{array}\right.$$

where *A*_max_ denotes the maximum AoI tolerated by the system. This model clearly indicates that once a ground-level vehicle experiences severe interference that causes its V2I transmission rate to fall below the threshold, its AoI will continue to increase linearly until it exceeds the tolerable limit, thereby triggering a system safety violation alarm.

Unlike V2I communication, which focuses on maintaining continuous information freshness, V2V communication primarily requires the delivery of a fixed-size data packet within a prescribed time interval. Following the C-V2X specifications, the PL is required to successfully deliver a CAM of size *ζ_j_* to all following vehicles within each 100-ms period. If any portion of the packet remains undelivered when the deadline expires, the transmission is considered a packet loss.

### 2.4. Problem Formulation and Delayed CSI-Aware Constraints

Based on the aforementioned physical and service models, the overall objective of the system is to jointly optimize the subchannel selection, communication mode, and transmit power of each platoon in the presence of complex and dynamic interference. Accordingly, the resulting problem can be formulated as a multi-objective mixed-integer nonlinear programming (MINLP) problem:

(9) $$\begin{array}{l} \underset{\beta ,\theta ,p}{\min}E\left[\sum_{j\in P}\left(\omega_{1}A_{j}^{t}-\omega_{2}\mathbb{P}\left\{\sum_{t=1}^{T}\sum_{k\in K}\min_{i}\{C_{j,i}^{t}[k]\}\Delta t\geq \zeta_{j}\right\}+\omega_{3}p_{j}^{t}\right)\right]\\ s.t.\;C1:\beta_{j,k}^{t},\theta_{j}^{t}\in \{0,1\},\forall j,k\\ \;\;\;\;\;\;\;C2:\sum_{k\in K}\beta_{j,k}^{t}\leq 1,\forall j,t\\ \;\;\;\;\;\;\;C3:0\leq p_{j}^{t}\leq p_{\max},\forall j,t\\ \;\;\;\;\;\;\;C4:{\tilde{h}}_{j}^{t}=h_{j}^{t-\tau } \end{array}$$

where E and P denote the expectation and probability operators, respectively. The three decision types are coupled through the link SINRs: subchannel reuse creates inter-platoon interference, the selected mode activates either the V2I or V2V requirement, and transmit power changes both the desired rate and the interference received by other platoons. The resulting rates also couple decisions over time through the AoI update and CAM deadline, making the objective and constraints nonseparable. The problem is NP-hard because, with powers and modes fixed, its interference-aware subchannel-assignment subproblem contains weighted graph coloring as a special case. In practical communication systems, channel estimation, data processing, and policy inference inevitably incur non-negligible latency. Therefore, when making resource allocation decisions at time slot (t), an agent cannot access the perfect instantaneous fast-fading channel state $h_{j}^{t}$ of the current slot. To capture this practical constraint, an information delay of *τ* = 2 ms is introduced into the proposed model. Specifically, the agent must generate its actions based on the outdated channel observation ${\tilde{h}}_{j}^{t}$, whereas the environment evaluates the resulting interference and reward using the actual current channel state $h_{j}^{t}$. This delayed-CSI constraint eliminates the conventional assumption of perfect prior channel information adopted by traditional optimization-based approaches and enables the proposed framework to more faithfully reflect practical vehicular communication environments.

## 3. AF-MARL: AoI- and Fairness-Aware Multi-Agent Resource Allocation

### 3.1. Multi-Agent Environment Modeling

The MARL problem involving multiple platoons considered in this study is formulated as follows:

(10) $$\begin{array}{l} \underset{U_{j}}{\max}\;L_{j}(U_{j})\\ s.t.\;C1:j\in P\\ \;\;\;\;\;\;C2:U_{j}\in U_{j} \end{array}$$

where $L_{j}(U_{j})=E[\sum_{t=0}^{\infty }\gamma^{t}R_{j}^{t+1}|s_{j}^{0}]$ and *U_j_* denote the policies of the *j* agent, $U_{j}$ represents the policy set of the *j* agent, and $R_{j}^{t+1}$ denotes the reward obtained by the *j* agent at the next time step after taking an action. The MINLP formulated above involves coupled discrete and continuous decision variables, together with constraints arising from imperfect state observations. Consequently, conventional convex optimization methods may struggle to obtain effective solutions within the millisecond-level decision latency required by vehicular networks. To address this challenge, we reformulate the problem as a decentralized partially observable Markov decision process (Dec-POMDP) and solve it within a MARL framework. In the proposed system, the PL of each platoon is regarded as an independent agent, which continuously interacts with the highly dynamic vehicular environment to learn an effective resource allocation policy.

For each agent *j* ∈ P, the Markov decision process at time slot *t* is defined by its local observation state, action space, and baseline reward function as follows:

State Space: To enable each agent to perceive the dynamics of the physical environment and make appropriate decisions, the local observation state (s) of agent *j* at time slot *t* incorporates comprehensive information about the network topology and service requirements. Specifically, the observation state includes the large-scale fading of the V2I and V2V links, the inevitably delayed observation of the fast-fading channel, the amount of co-channel interference experienced in the previous time slot, the current AoI level, the remaining time, and the amount of undelivered CAM data remaining in the current transmission cycle.

Action Space: At each time slot, the local actor of each agent produces a three-element normalized continuous action vector. The three elements control subchannel selection, communication-mode selection, and transmit power, respectively. Before execution, each element is clipped to the range from −1 to 1. For subchannel selection, this range is divided into *M* equal-width intervals, where *M* is the number of available resource blocks. A value falling in the *k*-th interval is assigned to the *k*-th resource block. The intervals are left-closed and right-open, except that the final interval includes the endpoint 1, thereby ensuring a unique resource-block assignment for every possible output. For communication-mode selection, a negative value of the second element selects the V2I mode, whereas a nonnegative value selects the V2V mode. The third element is linearly rescaled from the normalized range to the permitted transmit-power range from 1 to 30 dBm and is clipped at the two power limits. During training, exploration noise is added to the normalized actor output before clipping and action conversion. The deterministic conversion is applied only at the interface between the policy and the communication environment: the environment executes the resulting physical action, whereas the actor and critic networks retain the normalized continuous action representation during network optimization. Therefore, the discrete conversion is not included in the gradient calculation. The same conversion rule is used during training and evaluation, except that exploration noise is disabled during evaluation.

Reward Function: In conventional reinforcement learning settings, the local reward function is typically designed as the negative counterpart of the optimization objective. An agent receives positive rewards by successfully completing CAM transmission tasks over the V2V links, satisfying the minimum transmission-rate threshold of the V2I links to reduce AoI, and minimizing its own power consumption. However, when this conventional reward mechanism with a static power penalty is directly applied to the complex 3D heterogeneous environment described above, the multi-agent system tends to exhibit zero-sum-like behavior. To maximize their individual returns, vehicles with favorable channel conditions may transmit at high power, thereby generating severe interference and causing communication outages for vehicles experiencing unfavorable channel conditions. To address this issue, we reshape the reward function.

### 3.2. Fairness-Aware Reward Shaping

**Figure 2:**
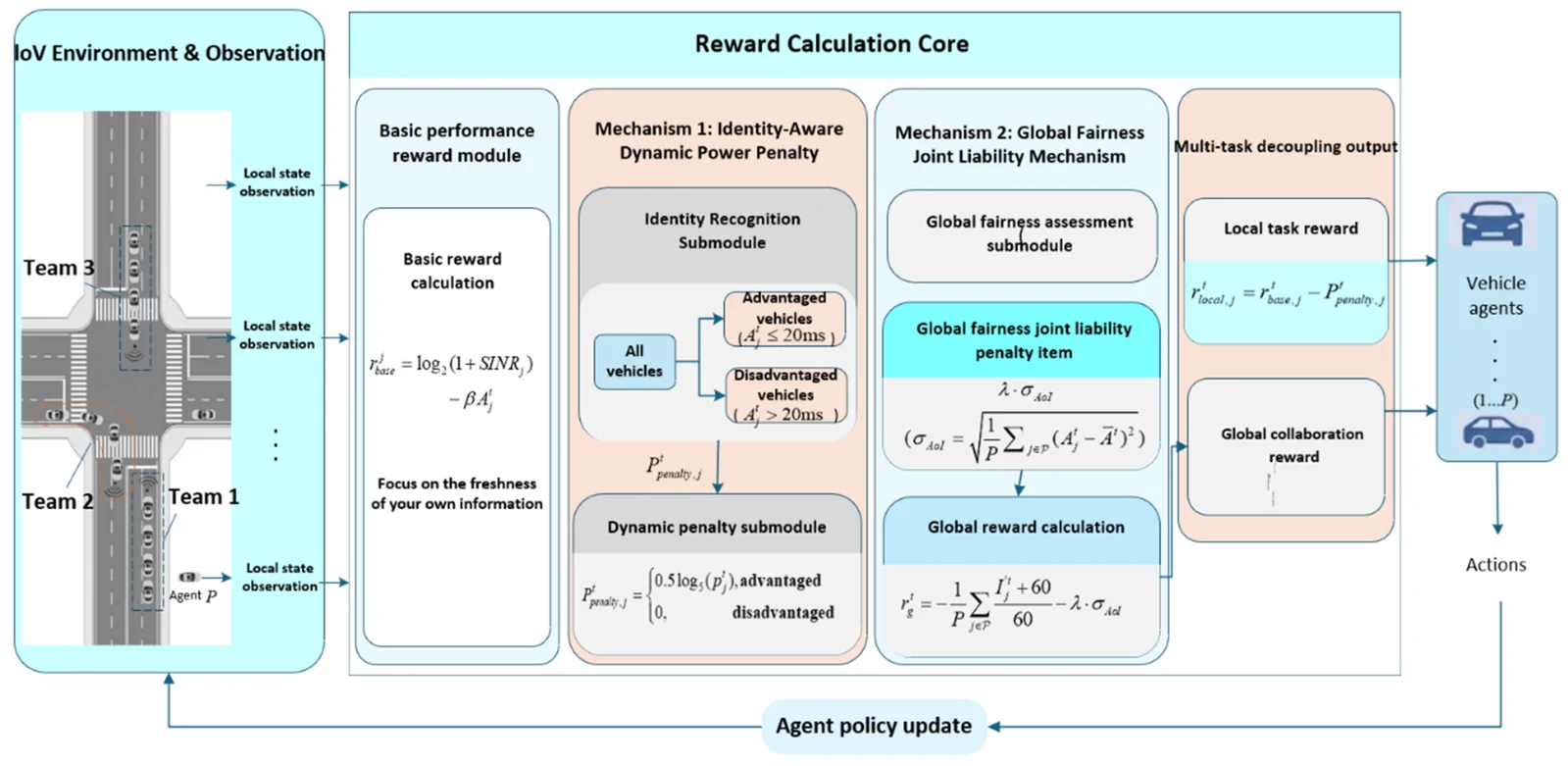
Architecture of the proposed AoI- and fairness-aware reward mechanism.

Figure 2 shows two complementary reward paths. The local path applies an AoI-dependent power cost to the task-specific rewards, whereas the global path combines cross-platoon AoI dispersion with system-wide interference. During centralized training, the corresponding local and global critics use these rewards jointly to guide the actor updates. The former depends only on the current AoI and transmit power; it does not directly identify the vehicle type, road level, channel class, or propagation condition.

AoI-dependent power-cost mechanism: Conventional resource-allocation algorithms commonly apply a fixed power cost to all agents. In the considered heterogeneous environment, however, the same power-cost incentive may restrict an agent even when its information has become stale. We therefore use the current AoI as a reward-gating variable. Specifically, the power-cost term imposed on agent *j* at time slot *t* is applied when the current AoI is no greater than the selected reward threshold and is removed when the AoI exceeds that threshold, as defined in Equation (11):

(11) $$P_{\mathrm{penalty},j}^{t}=\left\{\begin{array}{cc} \omega \log_{5}(p_{j}^{t}), & \mathrm{if}\;A_{j}^{t}\leq 20\;\mathrm{ms}\\ 0, & \mathrm{if}\;A_{j}^{t}>20\;\mathrm{ms} \end{array}\right.$$

where the coefficient controls the strength of the power cost. When the AoI does not exceed 20 ms, the logarithmic power penalty encourages platoons with sufficiently fresh information to reduce unnecessary transmit power. Once the AoI exceeds 20 ms, the penalty is removed to facilitate link recovery before the AoI reaches the 25 ms outage boundary. The 5 ms separation provides a guard interval for short-term channel fluctuations and delayed CSI. These thresholds are scenario-specific design choices rather than standardized safety limits.

Global Collective Fairness Penalty: Individual-level constraints alone are insufficient to ensure system-wide fairness. In a multi-agent system, the global critic is responsible for evaluating the overall cooperation among all agents. If the global reward only focuses on minimizing the average system-wide interference, the learning process may still improve the overall average performance at the expense of individual vehicles experiencing extremely unfavorable channel conditions. To equip the global critic with an explicit fairness-awareness capability, we introduce a collective fairness penalty into the global reward $r_{g}^{t}$, which is formulated based on the differences in AoI variations among platoons as follows:

(12) $$r_{g}^{t}=-\frac{1}{P}\sum_{j\in P}\frac{I_{j}^{\prime t}+60}{60}-\lambda \cdot \sigma_{\mathrm{AoI}}$$

(13) $$\sigma_{\mathrm{AoI}}=\sqrt{\frac{1}{P}\sum_{j\in P}{\left(A_{j}^{t}-{\bar{A}}^{t}\right)}^{2}}$$

where $I_{j}^{\prime t}=10\log_{10}(I_{j}^{t})$ denotes the normalized average system-wide interference, which constitutes the first term of the global reward; the second term represents the newly introduced fairness penalty; *σ_AoI_* denotes the standard deviation of the AoI values across all platoons at the current time slot; and *λ* is the balancing coefficient. The introduction of this mechanism enables the global critic to explicitly account for disparities in information freshness among platoons. If any disadvantaged platoon experiences a sharp increase in AoI due to severe physical obstruction, the system-wide AoI standard deviation *σ_AoI_* will increase accordingly, resulting in a substantial global penalty being imposed on all agents. Under this mechanism, agents are encouraged to shift from purely self-interested reward maximization toward more balanced and efficient allocation of system resources. This cooperative incentive ultimately discourages excessive transmit power and substantially reduces the overall co-channel interference floor in the environment.

### 3.3. Algorithm Architecture and Training Mechanism

The AF-MARL algorithm adopts a Modified Multi-Agent Deep Deterministic Policy Gradient with Task Decomposition (M-MADDPG-TDEC) architecture to address the multi-objective optimization problem arising from the conflicting requirements of V2V transmission deadlines and V2I information freshness in vehicular network resource allocation. The detailed procedure of the AF-MARL algorithm is presented in Algorithm 1. The training process incorporates an experience replay buffer and a Twin Delayed Deep Deterministic Policy Gradient (TD3) architecture to mitigate Q-value overestimation. At each time step, the agents interact with the environment and store the resulting transition tuple $\left(s^{t},a^{t},r_{\mathrm{loc}}^{t},r_{g}^{t},s^{t+1}\right)$ in the experience replay buffer D. During the network update phase, a mini-batch of *S* samples is randomly sampled from D to update the network parameters. The detailed network update process is described as follows:

To promote cooperation among agents and enable a global evaluation of the selected actions, two global Critic networks, $Q_{\psi_{1}}^{g_{1}}$ and $Q_{\psi_{2}}^{g_{2}}$, are deployed at the RSU. The two Critic networks are updated by minimizing the temporal-difference (TD) error. First, the global target value $y_{g}^{j}$ is calculated. To mitigate Q-value overestimation, the AF-MARL algorithm adopts the smaller output of the two target global Critic networks when computing the target Q-value:

(14) $$y_{g}^{j}=r_{g}^{j}+\gamma \min_{i}Q_{\psi_{i}^{\prime }}^{g_{i}}({s^{\prime }}^{j},{a^{\prime }}^{j})$$

where *γ* denotes the discount factor, while ${s^{\prime }}^{j}$ and ${a^{\prime }}^{j}$ represent the observation state and action at the next time step, respectively. Subsequently, the parameters *ψ_i_* of the global Critic networks are updated by minimizing the corresponding temporal-difference loss:

(15) $$T(\psi_{i})=\frac{1}{S}\sum_{j}\left\{{\left(Q_{\psi_{i}}^{g_{i}}(s^{j},a^{j})-y_{g}^{j}\right)}^{2}\right\}$$

Unlike conventional MADDPG, the AF-MARL algorithm decomposes the overall local reward of each agent into *M* independent subtask rewards and trains an independent local Critic network $Q_{\phi_{i,m}}^{i,m}$ for each subtask. The target value $y_{i,m}^{j}$ for the *m*-th subtask of agent *i* can be expressed as:

(16) $$y_{i,m}^{j}=r_{\mathrm{lo}c_{i,m}}^{j}+\gamma Q_{\phi_{i,m}^{\prime }}^{i,m}(s_{i}^{\prime j},a_{i}^{\prime j})$$

The parameters of the local subtask Critic network *ϕ_i_*_,*m*_ are updated as follows:

(17) $$T(\phi_{i,m})=\frac{1}{S}\sum_{j}\left\{{\left(Q_{\phi_{i,m}}^{i,m}(s_{i}^{j},a_{i}^{j})-y_{i,m}^{j}\right)}^{2}\right\}$$

The objective of the local Actor network $U_{\theta_{i}}$ is to learn an optimal policy that maximizes the global Q-value together with the sum of the Q-values of all local subtasks. Owing to the delayed policy update mechanism, the Actor network is updated less frequently than the Critic networks. For agent *i*, the policy gradient $\nabla J_{\theta_{i}}$ with respect to the Actor network parameters *θ_i_* combines the gradient feedback from the global Critic and all local subtask Critics, and is given by:

(18) $$\nabla J_{\theta_{i}}\approx \frac{1}{S}\sum_{j}\left\{\nabla_{\theta_{i}}U_{i}(a_{i}|s_{i}^{j})\nabla_{a_{i}}Q_{\psi_{1}}^{g_{1}}(s^{j},a^{j})+\sum_{m=1}^{M}\left[\nabla_{\theta_{i}}U_{i}(a_{i}|s_{i}^{j})\nabla_{a_{i}}Q_{\phi_{i,m}}^{i,m}(s_{i}^{j},a_{i}^{j})\right]\right\}$$

where when computing the policy gradient, only the output of the first global Critic network is used to evaluate the global Q-value. The AF-MARL algorithm adopts a soft-update strategy to update all target networks. Specifically, the parameters of each main network are gradually blended into its corresponding target network according to the update rate *τ*.

Target Global Critic Network Update:

(19) $$\psi_{i}^{\prime }\leftarrow \tau \psi_{i}+(1-\tau )\psi_{i}^{\prime }$$

Target Local Subtask Critic Network Update:

(20) $$\phi_{i,m}^{\prime }\leftarrow \tau \phi_{i,m}+(1-\tau )\phi_{i,m}^{\prime }$$

Target Local Actor Network Update:

(21) $$\theta_{i}^{\prime }\;\leftarrow \tau \theta_{i}\;+(1-\tau )\theta_{i}^{\prime }$$

**Figure 3:**
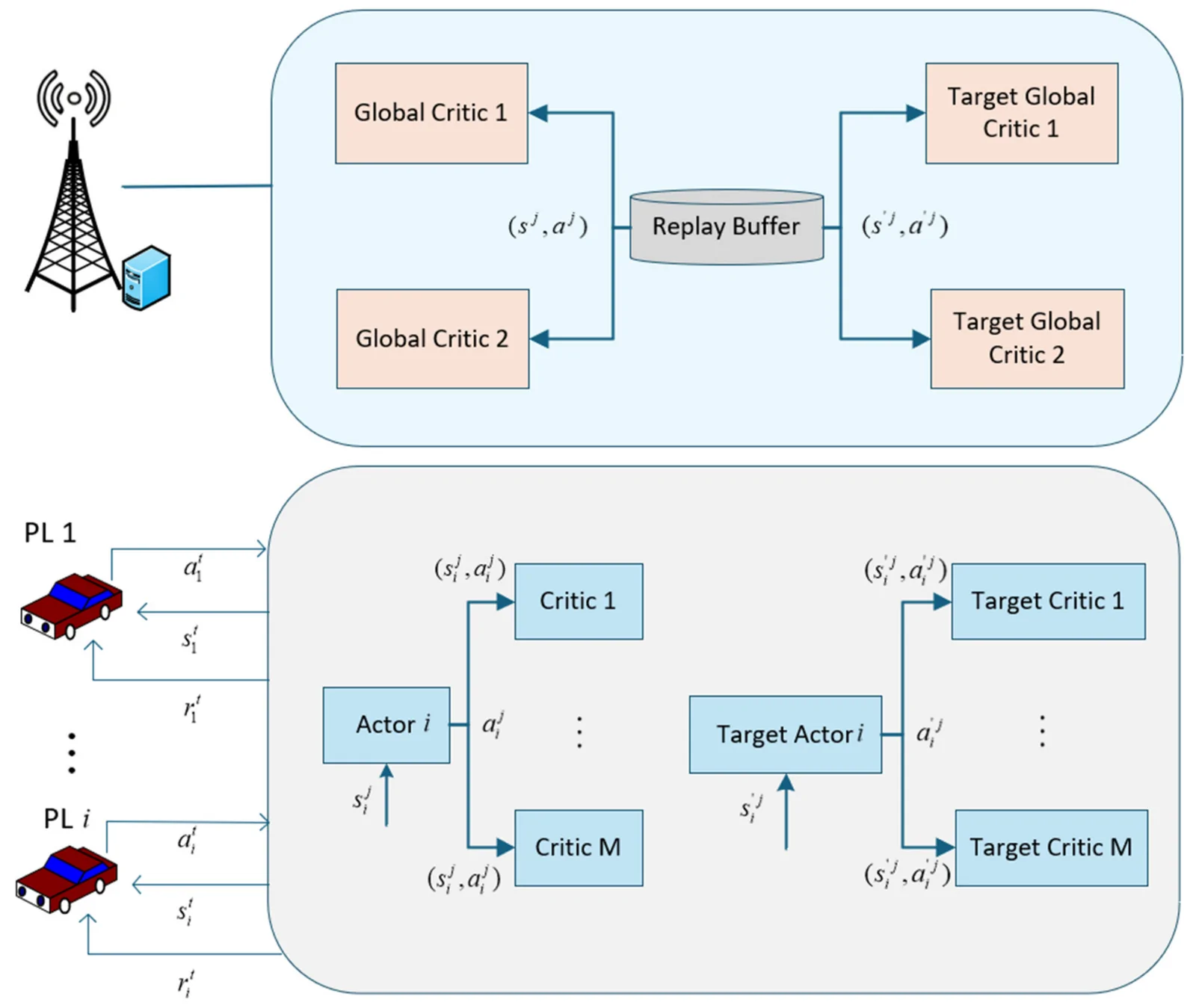
Training and resource-allocation architecture of AF-MARL.

As illustrated in Figure 3, the proposed architecture follows the centralized training and decentralized execution (CTDE) paradigm. Its key advantage lies in establishing a global–multi-local policy evaluation framework, in which the global Critic captures the overall system-level coordination performance, while multiple local Critic networks evaluate the task-specific performance of individual agents:
**Algorithm 1:** AoI- and Fairness-Aware Resource Allocation Algorithm Based on Improved Multi-Agent Reinforcement Learning1: **Initialize Input:** Initialize the parameters of the main global Critic networks $Q_{\psi_{1}}^{g_{1}}$, $Q_{\psi_{2}}^{g_{2}}$, target global Critic networks $Q_{\psi_{1}^{\prime }}^{g_{1}}$, $Q_{\psi_{2}^{\prime }}^{g_{1}}$, the Actor networks and local subtask Critic networks of all agents, their corresponding target networks *θ*′ and *ϕ*′, and the experience replay buffer D.2: **for** each episode **do**3:    Update the platoon positions and channel gains; reset the effective payload *ζ* and maximum delivery. deadline *T* to 100 ms;4:    **for** each time slot *t* **do**5:       **for** each agent *k* **do**6:          Observe the local state $s_{k}^{t}$ select action $a_{k}^{t}$
7:       **end for**8:       Calculate the power penalty $P_{\mathrm{penalty}}^{t}$, local reward $r_{\mathrm{loc}}^{t}$, standard deviation of the AoI values across all. platoons, and global reward $r_{g}^{t};$
9:       Store the transition tuple $\left(s^{t},a^{t},r_{\mathrm{loc}}^{t},r_{g}^{t},s^{t+1}\right)$ in the experience replay buffer D;10:     Randomly sample a mini-batch of *S* transition tuples $\left(s^{j},a^{j},r_{\mathrm{loc}}^{j},r_{g}^{j},s^{\prime }\right)$ form D11:     Calculate the global target Q-value $y_{g}^{j};$
12:     Update the global Critic network parameters *ψ_i_* by minimizing the mean squared error;13:     Soft-update the target network parameters $\psi_{i}^{\prime };$
14:     **if** *t* mod *d* **then**15:        **for** each agent *i* **do**16:            **for** each subtask *m* **do**17:               Calculate the target Q-value $y_{i,m}^{j};$
18:            Update the local subtask Critic network parameters *ϕ_i_*_,*m*_ by minimizing the MSE;19:            **end for**20:            Calculate the policy gradient and update the local Actor network;21:            **for** each subtask *m* **do**22:             Soft-update the target local subtask Critic network parameters $\phi_{i,m}^{\prime };$
23:            **end for**24:            Soft-update the target local Actor network parameters $\theta_{i}^{\prime };$
25:        **end for**26:     **end if**27:  **end for**28: **end for**


Local Multi-Task Evaluation Networks with Dynamic Penalties: To address the complexity arising from the simultaneous V2V and V2I communication requirements of each platoon PL, the proposed architecture decouples the conventional single local reward into multiple task-specific objectives and deploys multiple parallel local Critic networks at each PL. Specifically, the Task 1 Critic evaluates the reward associated with CAM message broadcasting, whereas the Task 2 Critic evaluates the reward associated with reducing AoI. In the proposed framework, the AoI-dependent power-cost mechanism introduced in Section 3.2 is incorporated into the local reward signals of both Task 1 and Task 2. Through this joint task decomposition and constraint integration, the Actor network receives fine-grained gradient guidance regarding transmit power control during policy updates, thereby encouraging vehicles with advantageous channel conditions to proactively reduce their transmit power and mitigate the interference imposed on disadvantaged platoons.

The reward function for Task 1 is defined as follows:

(22) $$r_{\mathrm{loc},j}^{t1}=-\omega_{1}\zeta_{j}^{\mathrm{rem}}/\zeta_{j}-\theta_{j}^{t}P_{\mathrm{penalty},j}^{t}$$

where *ω*_1_ denotes the weighting coefficient, and $\zeta_{j}^{\mathrm{rem}}$ represents the amount of CAM data remaining to be transmitted by agent *j*.

The reward function for Task 2 is defined as follows:

(23) $$r_{\mathrm{loc},j}^{t2}=\omega_{2}Y(C_{j,\mathrm{RSU}}^{t}-C_{\min})-\omega_{3}A_{j}^{t}-(1-\theta_{j}^{t})P_{\mathrm{penalty},j}^{t}$$

where $Y(x)={\{}_{0,\;x<0}^{V,\;x\geq 0}$ and *V* are positive constants representing the corresponding rewards, and *ω*_2_ and *ω*_3_ denote the weighting coefficients.

Global Evaluation Network with the Fairness Penalty Mechanism: At the RSU, a global Critic network with a system-wide view is deployed to centrally evaluate the overall performance of all agents. The global Critic collects the states and actions of all agents to assess their joint performance. Exploiting this capability, the AF-MARL algorithm incorporates the global fairness penalty mechanism into the global reward signal. Through backpropagation, the global Critic propagates the joint evaluation that accounts for both system-level interference and the standard deviation of AoI across all platoons to each agent. Consequently, each platoon is encouraged to optimize its individual reward while simultaneously accounting for the overall fairness of resource allocation across the system.

TD3-Based Delayed Update Mechanism for Mitigating Overestimation Bias: Under the severe 3D blockage and highly dynamic Doppler fading conditions considered in this study, abrupt variations in channel quality can easily lead to overestimation bias in the learned value functions. To address this issue, the proposed architecture adopts the core idea of TD3 by training two structurally identical global Critic networks in parallel at the RSU and taking the smaller of their outputs when computing the target Q-value. Meanwhile, the Actor network is updated less frequently than the Critic networks. This mechanism effectively improves the training stability of the agents under challenging and highly dynamic physical channel conditions.

### 3.4. Comparison with Existing AoI-Aware MARL Methods and Computational Complexity

Architecture and objectives: References [24,25,26] and AF-MARL all employ reinforcement-learning-based resource allocation for vehicular communication, but their optimization emphasis and critic organizations differ. Reference [24] focuses on the joint optimization of AoI, CAM delivery, power consumption, and energy efficiency. Reference [25] employs a cascaded multi-agent actor–critic design to improve critic evaluation for AoI- and CAM-related resource allocation. Reference [26] develops M-MADDPG-TDEC by combining local actors, twin global critics, task-specific local critics, TD3-based delayed updates, and task decomposition under centralized training with decentralized execution. AF-MARL directly inherits the learning architecture of [26]. Its methodological modifications are confined to the reward signals: the fixed power cost in the local task rewards is replaced by the AoI-dependent power cost in Equation (11), and the global interference reward is augmented by the cross-platoon AoI-dispersion term in Equations (12) and (13). Therefore, AF-MARL is a reward-level extension of [26], rather than a new actor–critic architecture.

Observation and action spaces: The recent AoI-aware approaches in [24,25,26] generally construct their observations from channel conditions, interference, AoI, and service-related information, while their actions represent combinations of spectrum allocation, communication-mode selection, transmit-power control, or energy-related decisions. In [26], each platoon leader observes local channel and interference information, its current AoI, the remaining CAM payload, and the transmission deadline, and then selects a subchannel, a V2I/V2V communication mode, and a transmit-power level. AF-MARL retains these physical action types and the corresponding categories of local observations. The difference is that the considered environment supplies delayed fast-fading observations and heterogeneous propagation conditions. The proposed reward modifications do not enlarge the state or action space; they only change how the consequences of the selected actions are evaluated during training.

Computational complexity of AF-MARL: Let *N* denote the number of platoon agents, *M* the number of local task critics per agent, *B* the mini-batch size, *d* the delayed actor-update interval, and *F_A_*, *F_L_*, and *F_G_* the forward-pass costs of one actor, one local task critic, and one global critic, respectively. For the inherited M-MADDPG-TDEC architecture, the leading critic-update cost is *O*(*B*(2*F_G_* + *NMF_L_*)), while actor-related evaluations occur every *d* updates and have order *O*((*BN*/*d*)(*F_A_* + *F_G_* + *MF_L_*)). Backpropagation changes only the constant factors of these expressions. Because AF-MARL introduces no additional actor or critic network relative to a dimension-matched implementation of [26], it has the same leading-order neural-network training complexity. Computing the *N* AoI-dependent power costs and the mean and standard deviation of the *N* AoI values adds only *O*(*N*) arithmetic per transition. During decentralized execution, each agent evaluates only its local actor, resulting in *O*(*F_A_*) inference complexity per decision, which is the same order as [26].

## 4. Simulations and Experimental Setup

This study conducts a comprehensive simulation-based performance evaluation of the AF-MARL algorithm. To ensure the reliability and comparability of the experimental results, the simulation environment is primarily constructed based on the urban intersection scenario defined in the 3GPP TR 36.885 specification. Based on this configuration, a simplified two-level three-dimensional scenario with a fixed ground-level excess-loss offset is used to evaluate the algorithm under controlled channel heterogeneity.

### 4.1. Simulation Environment

The simulation scenario is implemented in a Python 3.11 environment and executed on a computer equipped with an Intel Core i9-13900HX processor operating at 2.20 GHz, 16 GB of RAM, and the Windows 11 operating system. During the simulation, large-scale fading is assumed to vary slowly over a 100-ms interval, whereas small-scale fast fading evolves at a fine-grained time resolution of 1 ms. To accurately characterize the time-varying channel conditions induced by high-speed vehicle mobility, the small-scale fast-fading process is not generated as purely random white noise. Instead, an AR(1) model incorporating Doppler shifts determined by the actual relative velocities of the vehicles is employed to capture its temporal correlation. Furthermore, to evaluate the robustness of the AF-MARL algorithm under imperfect channel observations, a 2-ms CSI acquisition delay is imposed on the environment.

Regarding the hyperparameter settings, the AF-MARL algorithm adopts the TD3 mechanism, in which truncated Gaussian noise is added to the output of the target policy network to smooth the target policy. The delayed policy update interval is set to 2. To evaluate the effectiveness of the AF-MARL algorithm in mitigating short-term link interruptions and reducing the underlying interference floor, we compare it with conventional fully distributed reinforcement learning algorithms and include M-MADDPG-TDEC as the primary baseline. In all experiments, the number of RBs is fixed at three. The number of platoons, platoon size, and inter-vehicle spacing are varied to comprehensively evaluate the AoI and CAM transmission reliability of the algorithms under different network loads. The detailed simulation parameters are summarized in Table 1. In the reported simulations, three resource blocks are available; therefore, the first action element is divided into three equal-width intervals corresponding to RB 1, RB 2, and RB 3. The communication-mode boundary is set to zero, and the power-control element is rescaled to the range from 1 to 30 dBm. In the principal five-platoon experiments, P1–P3 are elevated-road platoons and P4–P5 are ground-level platoons. The maximum AoI is 100 ms, and the transmit-power range is 1–30 dBm. Because the original source code and complete training configuration are unavailable, the unreported value of *C*_min_, the reward coefficients including λ and *V*, the exploration- and target-policy-noise settings, the target-network update rate, and the shadowing parameters cannot be independently verified.

### 4.2. Simulation Results and Analysis

**Figure 4:**
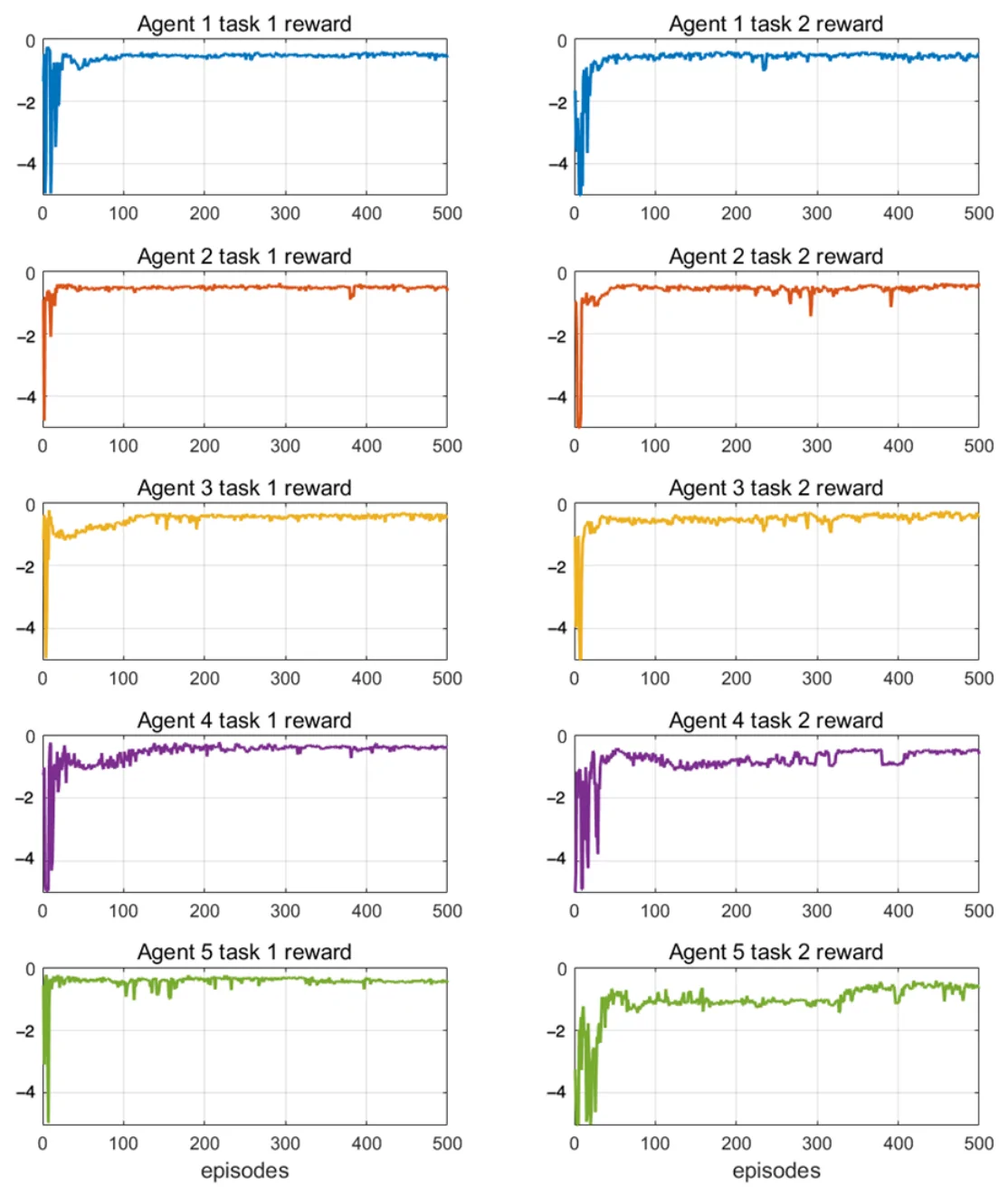
Convergence of agent-specific subtask rewards.

Figure 4 illustrates the convergence performance of the subtask rewards for each agent when the number of members in each platoon is set to 4 and the intra-platoon spacing is 25 m. As shown in the figure, the Task 2 rewards of Agents 4 and 5 require more training iterations to converge than those of Agents 1–3. This difference is mainly attributed to the additional NLoS penetration loss experienced by Platoons 4 and 5 in the heterogeneous physical environment, which deteriorates their channel conditions and increases the difficulty of learning an optimal resource allocation policy. In addition, the reward curves exhibit certain fluctuations during training due to the time-varying channel fading induced by vehicle mobility. Nevertheless, all agents achieve stable convergence within fewer than 100 training episodes, indicating that the AF-MARL algorithm can effectively adapt to the dynamic channel environment and learn policies that maximize the corresponding subtask rewards.

**Figure 5:**
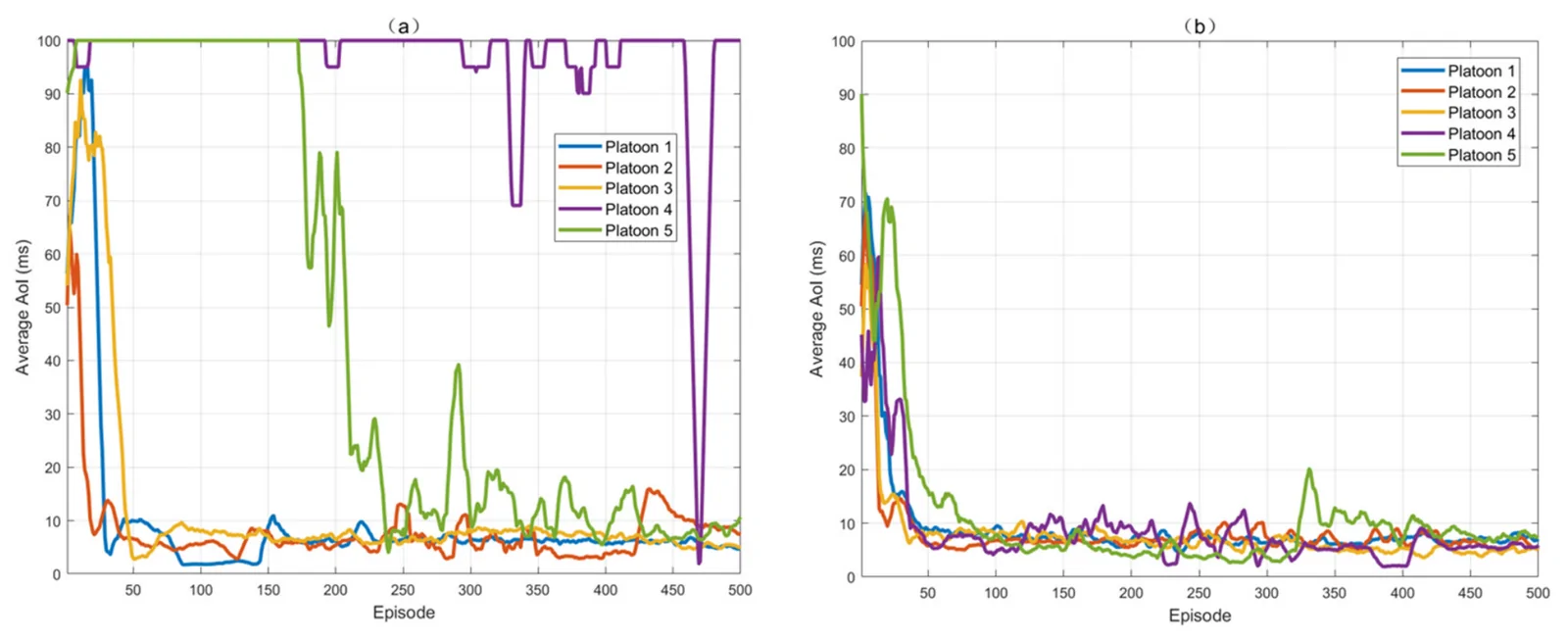
AoI convergence of individual platoons under different algorithms: (**a**) average AoI of each platoon over training episodes under the M-MADDPG-TDEC baseline algorithm; (**b**) average AoI of each platoon over training episodes under the proposed AF-MARL algorithm.

To evaluate the effectiveness of the AF-MARL algorithm in addressing communication unfairness in the 3D heterogeneous scenario, Figure 5 presents the convergence of the average AoI of the five platoons over the training episodes for both the AF-MARL algorithm and the baseline algorithms. The results illustrate the convergence characteristics of the AF-MARL algorithm from a system-level perspective and provide a direct comparison of its ability to maintain information freshness across different platoons.

Figure 5a presents the AoI convergence curves of the baseline algorithm, M-MADDPG-TDEC. It can be observed that, under the severe physical blockage and heterogeneous channel conditions, the system suffers from link interruptions and significant resource allocation unfairness. Specifically, the AoI values of Platoons 1–3 decrease relatively rapidly and eventually stabilize below 10 ms, mainly because these platoons are located in favorable physical positions and thus benefit from intrinsically better channel conditions. In contrast, Platoons 4 and 5, which experience disadvantaged channel conditions, encounter severe communication bottlenecks. The AoI of Platoon 4 remains at the maximum penalty threshold of 100 ms throughout almost the entire 500 training episodes, indicating a persistent interruption of its V2I link. Although the AoI of Platoon 5 decreases after remaining at 100 ms for approximately 150 episodes, its convergence remains highly unstable with substantial fluctuations. These results indicate that the conventional static power-penalty mechanism in M-MADDPG-TDEC fails to effectively address the resource competition caused by heterogeneous channel conditions. Agents with favorable channels tend to increase their transmit power to maximize their individual communication performance and local rewards, thereby raising the interference floor in the system. Consequently, platoons already suffering from severe channel degradation are more vulnerable to interference, resulting in prolonged or intermittent V2I link interruptions and a substantial increase in AoI. This phenomenon demonstrates the inability of the baseline algorithm to adequately protect disadvantaged platoons and maintain fairness in the 3D heterogeneous environment.

In contrast, as shown in Figure 5b, the AF-MARL algorithm exhibits substantially improved fairness and training stability. As observed from the figure, after an initial policy exploration phase of approximately 50 episodes, the AoI values of all five platoons, including the previously disconnected Platoons 4 and 5, rapidly converge to the safe range of 5–10 ms. The AoI values then remain stable over the subsequent training episodes, without significant spikes or link interruptions. This improvement can be attributed to the joint effect of the AoI-dependent power-cost mechanism and the global fairness penalty mechanism. When the AoI of a platoon with favorable channel conditions falls within the safe range, the dynamic power penalty encourages the corresponding agent to proactively reduce its transmit power. This power backoff does not compromise its information freshness, while effectively reducing the interference imposed on other platoons. Meanwhile, the global fairness penalty further discourages resource allocation policies that sacrifice disadvantaged platoons for improved system-level performance. As a result, the AF-MARL algorithm effectively suppresses excessive interference from advantageous platoons and creates more favorable communication conditions for disadvantaged ground platoons, thereby achieving a more balanced and stable AoI performance across the entire system.

**Figure 6:**
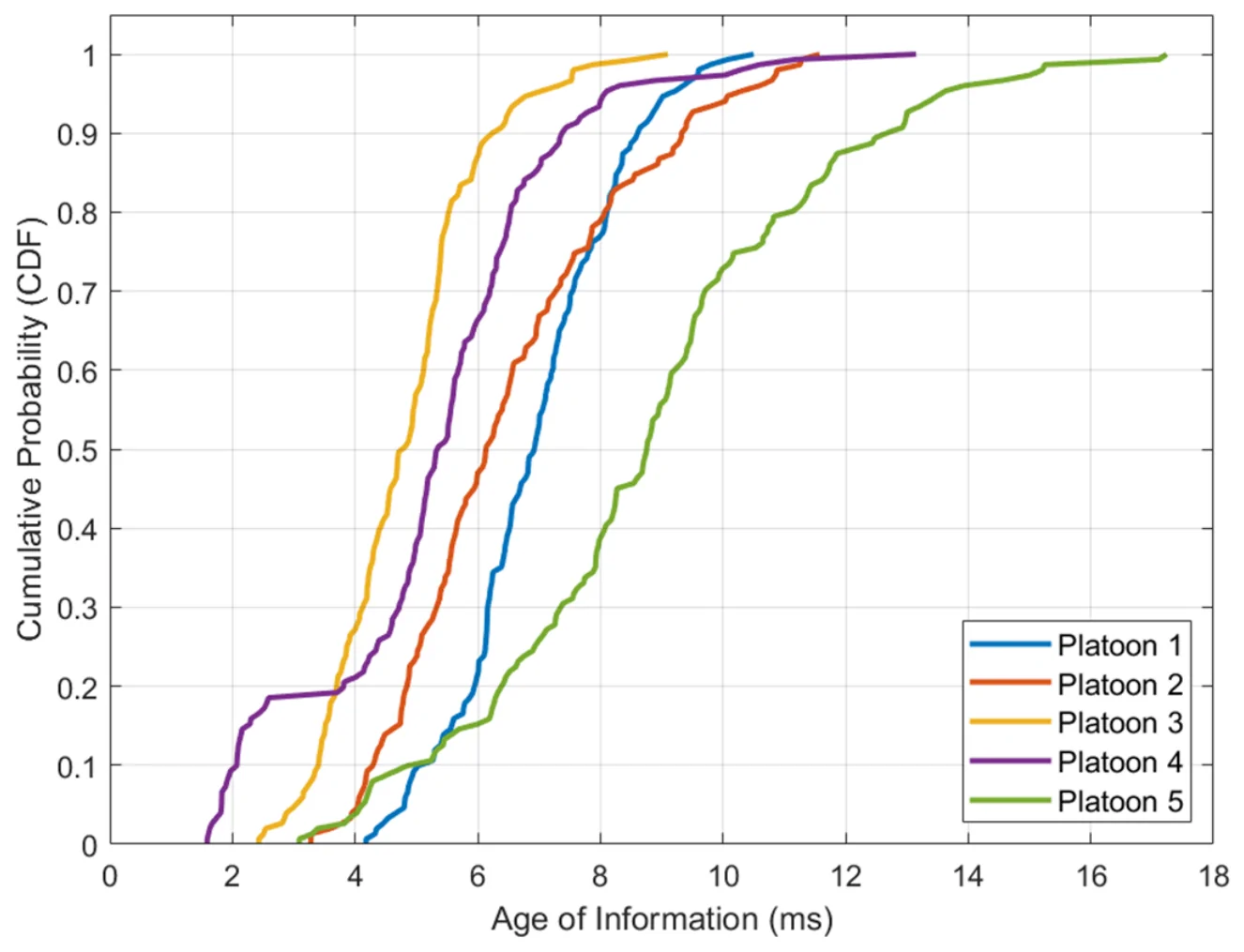
Empirical cumulative distributions of AoI for individual platoons.

To further evaluate the reliability and fairness of the AF-MARL algorithm after policy convergence, Figure 6 presents the cumulative distribution function (CDF) of the AoI for the five platoons. As shown in the figure, the AoI of all five platoons is effectively bounded below 18 ms, and the CDF of each platoon reaches 1 at approximately 18 ms. Even for Platoon 5, which operates under severe ground-level blockage and disadvantaged channel conditions, the CDF exhibits only a slight tail while still reaching 1 within the 18-ms range. This indicates that the AF-MARL algorithm effectively prevents prolonged AoI accumulation and avoids communication interruptions. The slight tail observed for Platoon 5 can be attributed to the highly dynamic vehicular environment, where vehicle mobility and time-varying channel fading introduce unavoidable fluctuations in communication performance. Nevertheless, the five CDF curves remain highly concentrated, demonstrating a relatively consistent AoI distribution across different platoons. This result further confirms that the combination of the global fairness penalty and AoI-dependent power-cost mechanism enables advantageous platoons to proactively reduce excessive transmit power, thereby mitigating interference experienced by disadvantaged platoons. Consequently, the AF-MARL algorithm avoids converging to a locally optimal policy that improves the performance of strong links at the expense of weak links, and achieves a more reliable and equitable allocation of communication resources.

**Figure 7:**
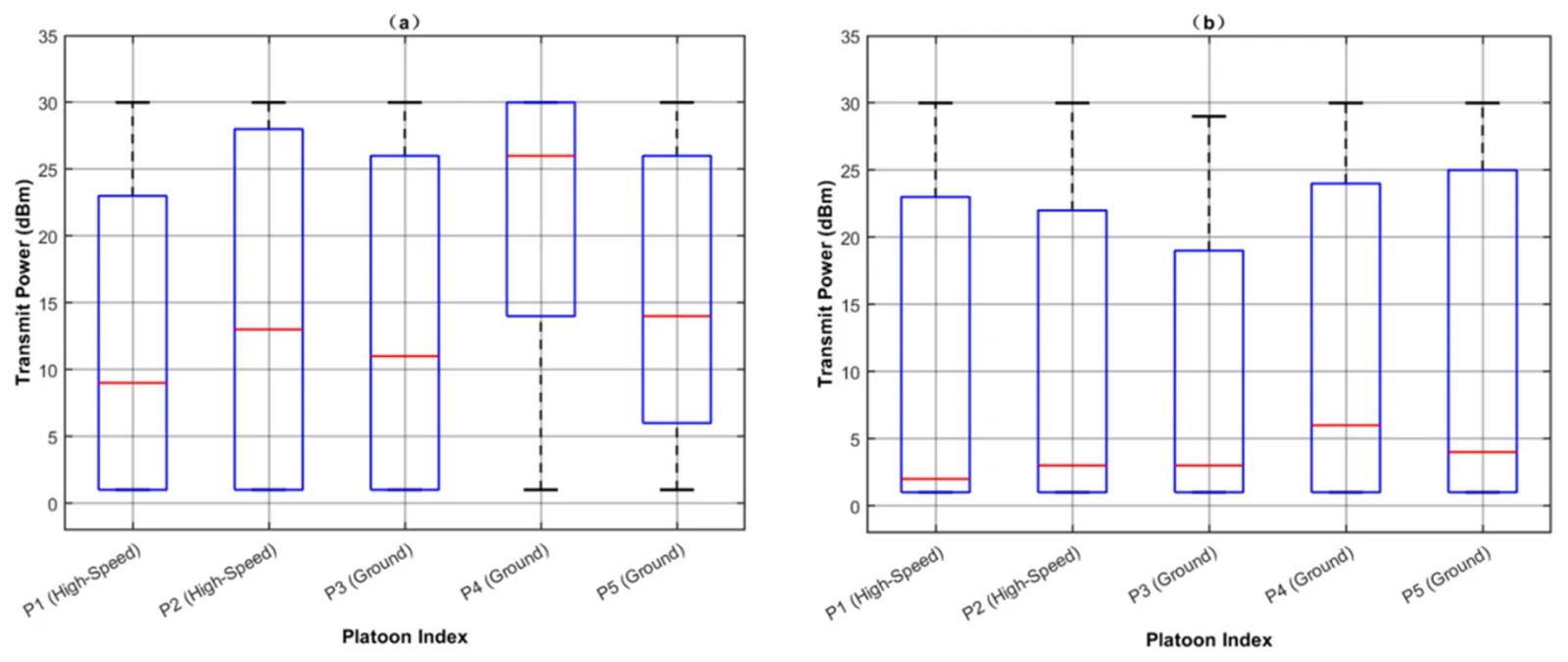
Transmit-power distributions of individual platoons under the baseline and AF-MARL algorithms: (**a**) boxplots of the transmit power of the five platoons under the M-MADDPG-TDEC baseline algorithm; (**b**) corresponding boxplots under the proposed AF-MARL algorithm. Red horizontal lines indicate the medians, and boxes represent the interquartile ranges.

To further investigate the resource allocation strategy and energy-efficiency performance at the microscopic level, Figure 7 presents the boxplot distributions of the actual transmit power of each platoon after the policies have converged, comparing the baseline algorithm with the AF-MARL algorithm. Figure 7a shows the transmit-power distribution obtained by the baseline algorithm. It can be observed that the agents generally adopt relatively high transmit power levels. Due to the uniform static power penalty and the lack of an effective cooperative power-backoff mechanism, the LoS-dominant Platoons P1 and P2 tend to frequently transmit at high power to maximize their own throughput. This selfish power allocation behavior substantially increases the co-channel interference in the underlying environment, forcing the ground platoons to further increase their transmit power to maintain their basic communication links. In particular, the median transmit power of the disadvantaged ground Platoon P4 reaches approximately 26 dBm, while its upper quartile approaches the maximum power limit of 30 dBm. This high-interference and high-power operating state indicates that the disadvantaged platoons must expend considerable transmission power merely to compensate for the unfavorable channel conditions and external interference. It also provides a microscopic explanation for the prolonged data-update stagnation and elevated AoI observed for disadvantaged platoons in the corresponding AoI convergence results.

In contrast, as shown in Figure 7b, the AF-MARL algorithm exhibits a more energy-efficient and cooperative power allocation pattern. The transmit power of all platoons decreases substantially, with the median power of all five platoons falling below 6 dBm. In particular, the median transmit power of the elevated Platoons P1 and P2 decreases to approximately 2–3 dBm, while their transmit power remains close to the minimum level of 1 dBm for most of the operating time. These results provide direct evidence that the proposed dynamic power penalty effectively encourages platoons with favorable channel conditions to proactively reduce their transmit power without compromising communication freshness. The resulting power backoff also alleviates co-channel interference, creating more favorable transmission conditions for disadvantaged platoons and promoting more energy-efficient and cooperative resource allocation across the system.

Furthermore, the proactive power reduction in platoons with favorable channel conditions effectively lowers the co-channel interference floor in the physical environment, providing disadvantaged ground platoons with a cleaner communication environment. As a result, these platoons no longer need to maintain persistently high transmit power to overcome excessive interference. Instead, they can complete V2I status updates with substantially lower transmit power while maintaining reliable communication performance. This result further demonstrates the effectiveness of the proposed fairness-aware power control mechanism in simultaneously reducing interference and improving the energy efficiency of disadvantaged platoons.

**Figure 8:**
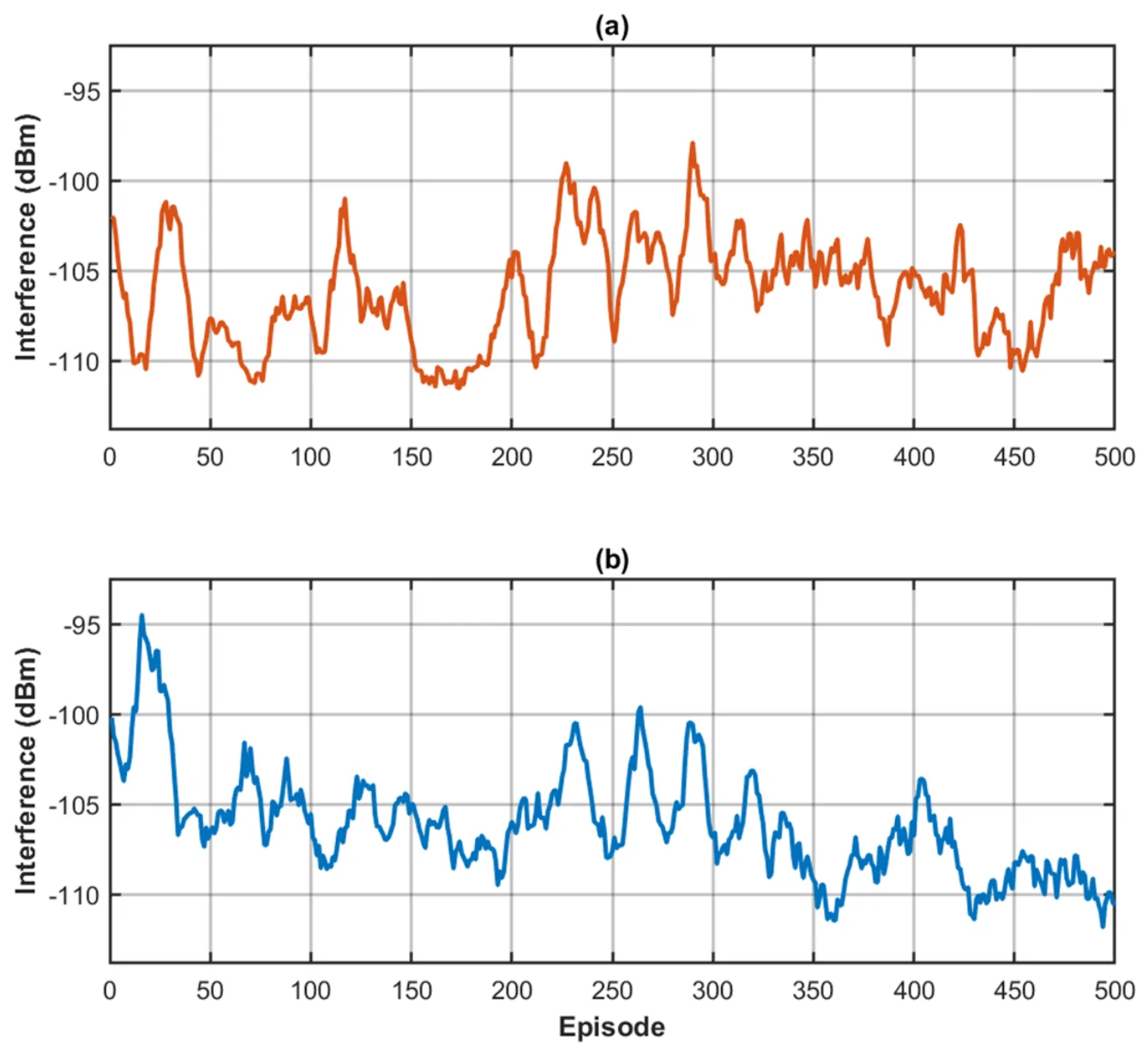
Interference-floor comparison between the baseline and AF-MARL algorithms: (**a**) interference level over training episodes under the M-MADDPG-TDEC baseline algorithm; (**b**) interference level over training episodes under the proposed AF-MARL algorithm.

To verify the effectiveness of power control in improving the communication environment, Figure 8 compares the actual physical interference floors achieved by the AF-MARL algorithm and the baseline algorithm. As shown in Figure 8a, the baseline algorithm maintains a relatively high interference floor, frequently exceeding −100 dBm and exhibiting persistent fluctuations around a high level. This behavior can be attributed to the lack of an effective cooperative power-backoff mechanism, which allows platoons with favorable channel conditions to transmit at relatively high power, resulting in a persistent high-interference operating state. In contrast, as shown in Figure 8b, the interference level of the AF-MARL algorithm exhibits a gradual downward trend after the initial exploration phase, despite certain fluctuations caused by the dynamic channel environment. After approximately 350 training episodes, the interference floor remains below −105 dBm for an extended period. These results demonstrate that the proposed dynamic power penalty and global fairness penalty effectively guide advantageous platoons to proactively reduce their transmit power, thereby suppressing the overall interference floor and providing a cleaner communication environment for disadvantaged platoons.

**Figure 9:**
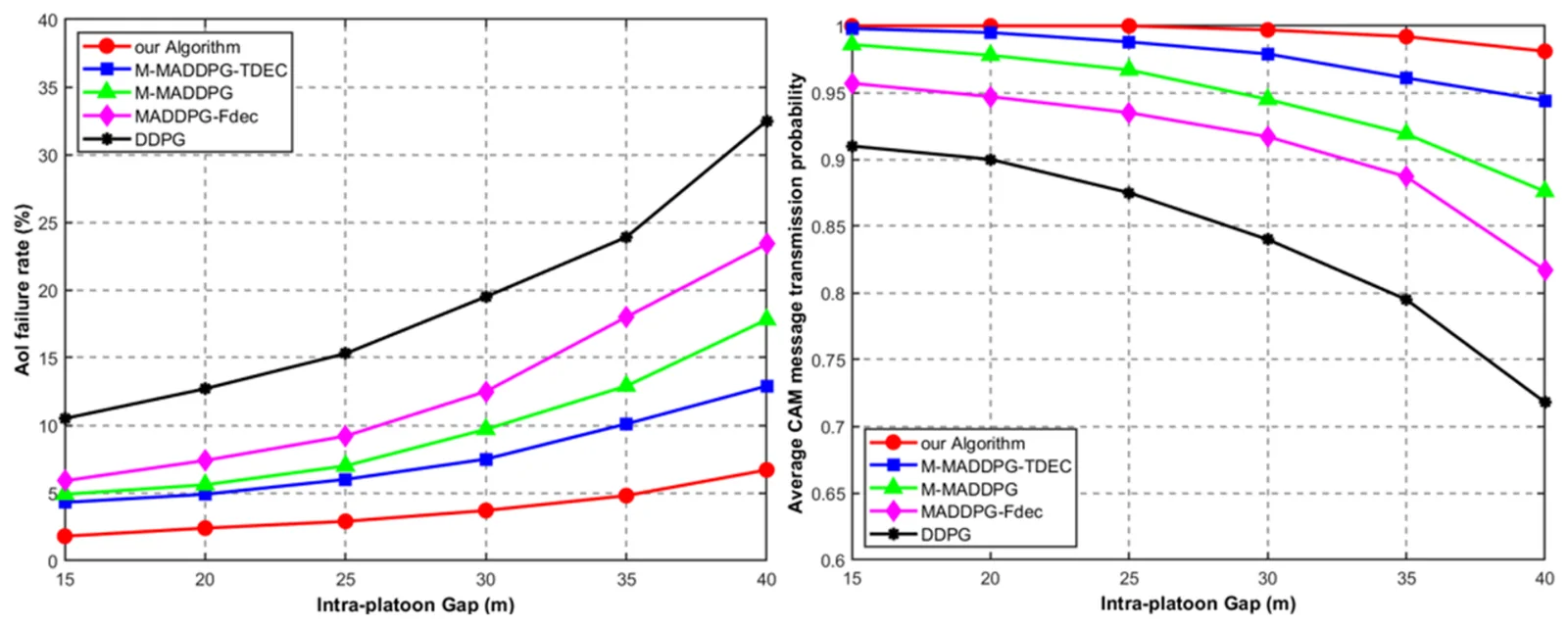
AoI outage rate and CAM transmission reliability versus intra-platoon spacing.

As shown in Figure 9, we evaluate the AoI outage rate and CAM transmission reliability as the intra-platoon spacing increases. An AoI above 25 ms is counted as an outage, whereas the 20 ms reward threshold is an earlier control trigger. The 5 ms margin allows the power penalty to be removed before an outage is recorded. It can be observed that the performance of all algorithms gradually deteriorates as the intra-platoon spacing increases. When the spacing reaches 40 m, the AoI outage rate of the baseline algorithm increases to approximately 13%, whereas that of the AF-MARL algorithm remains below 7% throughout the considered range. Regarding CAM transmission reliability, the other algorithms exhibit a noticeable degradation as the intra-platoon spacing increases. In contrast, the curve of the AF-MARL algorithm remains nearly flat, maintaining a success rate above 98% even at an intra-platoon spacing of 40 m. These results demonstrate that the AF-MARL algorithm effectively coordinates the resource competition between V2I and V2V communications. Consequently, it can maintain reliable communication performance even when the spatial span within a platoon becomes relatively large, demonstrating strong robustness to changes in network topology.

**Figure 10:**
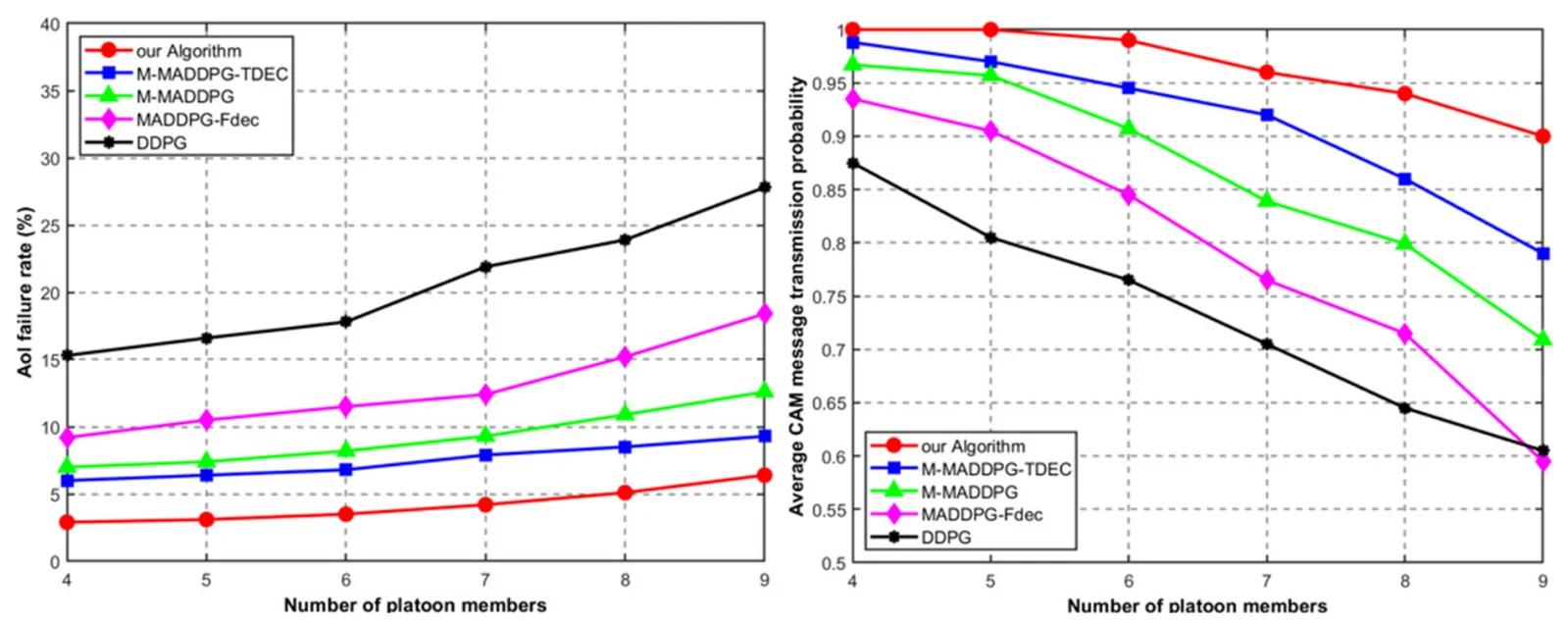
AoI outage rate and CAM transmission reliability versus platoon size.

Since the number of resource blocks remains fixed in the system, variations in platoon size have a significant impact on the resource allocation process. Figure 10 illustrates the performance trends of different algorithms in terms of AoI outage rate and CAM transmission reliability as the platoon size increases. It should be noted that, as the number of platoon members increases from 4 to 9, the PL must deliver CAM messages to more follower vehicles within the prescribed deadline. Consequently, the concurrent communication load in the system increases substantially, leading to more intense competition for the limited spectrum resources and higher co-channel interference.

The AoI outage rate curves show that the outage rate of all algorithms inevitably increases as the platoon size expands. Among them, DDPG and the fully decentralized MADDPG (MADDPG-Fdec) algorithm lack effective task decomposition and global coordination mechanisms, resulting in limited adaptability to dynamic environmental changes. Consequently, their outage rates deteriorate rapidly as the number of platoon members increases, reaching approximately 27.6% and 18%, respectively, at the largest platoon size. The M-MADDPG-TDEC baseline [26], which incorporates a task-decomposition architecture, achieves relatively better performance. Nevertheless, when the platoon size reaches nine vehicles, its AoI outage rate still approaches 10%. In contrast, the AF-MARL algorithm maintains the outage rate at only 6.2% even with nine platoon members, demonstrating superior scalability and robustness under increasing communication loads.

For the CAM transmission reliability of the V2V links, an increase in the number of platoon members leads to higher network density and, consequently, a greater probability of signal collisions. As the communication load increases, the transmission success rates of DDPG and MADDPG-Fdec decline sharply, eventually remaining at only approximately 60%. Although the M-MADDPG-TDEC baseline performs relatively well under lower loads, its reliability also decreases significantly to approximately 79% when the platoon size reaches nine vehicles. In contrast, the AF-MARL algorithm exhibits the most gradual decline and maintains a high CAM transmission reliability of approximately 90% even at the maximum platoon size. This performance advantage under heavy communication loads further demonstrates the effectiveness of the proposed task-aware and fairness-aware resource allocation mechanism in coordinating V2I and V2V communication demands under constrained spectrum resources.

Overall, as the platoon size increases and the network density becomes higher, the AF-MARL algorithm effectively coordinates the resource competition between V2I and V2V links. By enabling limited spectrum and power resources to be allocated more efficiently and fairly across multiple communication tasks and platoons, the AF-MARL algorithm maintains reliable system-wide communication performance under increasingly heavy network loads. These results demonstrate its robustness and scalability in the considered 3D heterogeneous vehicular environment.

## 5. Conclusions

This study investigated joint communication-resource allocation for timely sensing-data dissemination in a simplified two-level vehicular scenario. To represent practical C-V2X operating conditions, we modeled the physical blockage caused by elevated and ground-level roads, mobility-induced Doppler effects, and delayed and imperfect CSI. The joint selection of subchannels, communication modes, and transmit power was formulated as a partially observable decision problem. We then developed AF-MARL, which combines an AoI-dependent power-cost mechanism with a cross-platoon AoI-dispersion penalty. The former encourages platoons with favorable channels to avoid unnecessary high-power transmission, whereas the latter prevents system-level optimization from sacrificing disadvantaged platoons. Simulation results showed that AF-MARL reduced the interference floor from approximately −105 dBm to −110 dBm and eliminated persistent AoI outages for platoons affected by severe blockage. Under increasing intra-platoon spacing and platoon size, AF-MARL also maintained reliable CAM delivery over V2V links and fresh vehicle-status updates over V2I links. These findings show improved information freshness and CAM delivery relative to the considered baselines under the implemented 20 ms reward-gating threshold and fixed reward-weight configuration. They do not establish the optimality of the selected threshold, the robustness of the reward parameters, or the stability of the hard-switching mechanism in Equation (11). Nevertheless, the present evaluation is simulation based and considers a single-RSU intersection with a fixed 10 dB excess-loss abstraction. Therefore, the results do not establish performance under site-specific geometry-dependent blockage. Future work will incorporate position-dependent LoS/NLoS conditions and building-aware or measurement-driven channel models.

## Figures and Tables

**Table 1:** Simulation parameter table.

Vehicle and Environment Parameters	Value
Carrier frequency	5.9 GHz
Number of resource blocks	3
Bandwidth per RB	180 kHz
Number of vehicles	20–45
Platoon size	4–9
Elevated-vehicle speed/antenna height	19–25 m/s, 10 m
Ground-vehicle speed/antenna height	4–10 m/s, 1.5 m
Intra-platoon spacing	15, 20, 25, 30, 35, 40 m
RSU height	25 m
Maximum transmit power	30 dBm
Noise power	−114 dBm
CAM message size	4000 bytes
CAM transmission deadline	100 ms
V2I path-loss model	128.1 + 20log_10_(*f_c_*/2.0) + 37.6log_10_(*d*_3*d*_/1000)
NLoS penetration loss	10 dB
CSI acquisition delay	2 ms
Small-scale fading	Rayleigh fading
Neural Network Parameters	Value
Mini-batch size	64
Local Actor hidden layers/dimensions	2/1024, 512
Local Critic hidden layers/dimensions	2/512, 256
Global Critic hidden layers/dimensions	3/1024, 512, 256
Critic/Actor learning rate	0.002/0.0005
Discount factor	0.99
Number of episodes	500
Iterations per episode	100
Replay buffer size	50,000

## Data Availability

Data are contained within the article.

## References

[1] Hildebrand B., Baza M., Salman T., Tabassum S., Konatham B., Amsaad F., Razaque A. (2023). A comprehensive review on blockchains for Internet of Vehicles: Challenges and directions. Comput. Sci. Rev..

[2] Khan Z., Koubaa A., Benjdira B., Boulila W. (2023). A game theory approach for smart traffic management. Comput. Electr. Eng..

[3] Elsagheer Mohamed S.A., AlShalfan K.A. (2021). Intelligent traffic management system based on the internet of vehicles (IoV). J. Adv. Transp..

[4] Zhu S., Liu C., Zhu H., Qiao R., Chen H., Zhang H. (2025). Multi-objective optimization task offloading decision for intelligent transportation system in cloud edge collaborative computing scenario. Wirel. Netw..

[5] Kumar V., Ali R., Sharma P.K. (2024). IoV-6G+: A secure blockchain-based data collection and sharing framework for Internet of vehicles in 6G-assisted environment. Veh. Commun..

[6] Tang L., Wang L., Fang D., Chen Q. (2025). Dynamic slice resource management and information synchronization strategy in IoV based on digital twin. IEEE Trans. Intell. Transp. Syst..

[7] Ahmed M., Liu J., Mirza M.A., Khan W.U., Al-Wesabi F.N. (2023). MARL based resource allocation scheme leveraging vehicular cloudlet in automotive-industry 5.0. J. King Saud Univ.-Comput. Inf. Sci..

[8] Ullah I., Singh S.K., Adhikari D., Khan H., Jiang W., Bai X. (2025). Multi-Agent Reinforcement Learning for task allocation in the Internet of Vehicles: Exploring benefits and paving the future. Swarm Evol. Comput..

[9] Huang B., Fan X., Zheng S., Chen N., Zhao Y., Huang L., Gao Z., Chao H.-C. (2025). Collaborative sensing-aware task offloading and resource allocation for integrated sensing-communication-and computation-enabled internet of vehicles (iov). Sensors.

[10] Lin Z., Pan H., Wang Y. (2025). Optimization of Spectrum Resource Allocation for Vehicle Platoon in V2X Networks Based on Deep Reinforcement Learning. IEEE Trans. Veh. Technol..

[11] Wang L., Liang H., Zhao D. (2023). Deep-reinforcement-learning-based computation offloading and power allocation within dynamic platoon network. IEEE Internet Things J..

[12] Wang P., Di B., Zhang H., Bian K., Song L. (2019). Platoon cooperation in cellular V2X networks for 5G and beyond. IEEE Trans. Wirel. Commun..

[13] Peng H., Li D., Ye Q., Abboud K., Zhao H., Zhuang W., Shen X. (2017). Resource allocation for cellular-based inter-vehicle communications in autonomous multiplatoons. IEEE Trans. Veh. Technol..

[14] Zeng T., Semiari O., Saad W., Bennis M. (2019). Joint communication and control for wireless autonomous vehicular platoon systems. IEEE Trans. Commun..

[15] Wang R., Wu J., Yan J. (2018). Resource allocation for D2D-enabled communications in vehicle platooning. IEEE Access.

[16] Viadero-Monasterio F., Meléndez-Useros M., Jiménez-Salas M., Boada M.J.L. (2025). Fault-Tolerant Robust Output-Feedback Control of a Vehicle Platoon Considering Measurement Noise and Road Disturbances. IET Intell. Transp. Syst..

[17] Yang H., Xie X., Kadoch M. (2019). Intelligent resource management based on reinforcement learning for ultra-reliable and low-latency IoV communication networks. IEEE Trans. Veh. Technol..

[18] Fu X., Yuan Q., Luo G., Cheng N., Li Y., Wang J., Liao J. (2024). HierNet: A hierarchical resource allocation method for vehicle platooning networks. IEEE Internet Things J..

[19] Zhang H., Liang H., Wang L., Yao Y., Lin B., Zhao D. (2024). Joint resource allocation and security redundancy for autonomous driving based on deep reinforcement learning algorithm. IET Intell. Transp. Syst..

[20] Xu Y., Zhu K., Xu H., Ji J. (2023). Deep reinforcement learning for multi-objective resource allocation in multi-platoon cooperative vehicular networks. IEEE Trans. Wirel. Commun..

[21] Zhao L., Feng Y., Hawbani A., Xu L., Liu Z., Bi Y. (2025). Optimized resource allocation in vehicle edge computing through platoon collaboration. IEEE Internet Things J..

[22] Chen C., Jiang J., Lv N., Li S. (2020). An intelligent path planning scheme of autonomous vehicles platoon using deep reinforcement learning on network edge. IEEE Access.

[23] Nan Z., Jia Y., Ren Z., Chen Z., Liang L. (2021). Delay-aware content delivery with deep reinforcement learning in internet of vehicles. IEEE Trans. Intell. Transp. Syst..

[24] Zheng Y., Nguyen L.D., Duong T.Q. (2026). Multi-Agent Deep Reinforcement Learning for Optimal Resource Allocation in AoI-Aware Energy-Efficient Platoon-Based C-V2X Networks. IEEE Internet Things J..

[25] Narayanasamy I., Rajamanickam V. (2024). A cascaded multi-agent reinforcement learning-based resource allocation for cellular-V2X vehicular platooning networks. Sensors.

[26] Liu X., Liu H., Zheng K., Liu J., Taleb T., Shiratori N. (2025). AoI-Minimal Clustering, Transmission and Trajectory Co-Design for UAV-Assisted WPCNs. IEEE Trans. Veh. Technol..

[27] Zanella A., Bazzi A., Pasolini G., Masini B.M. (2013). On the impact of routing strategies on the interference of ad hoc wireless networks. IEEE Trans. Commun..

[28] Sun M., Xu J., Wang J. (2026). State-Aware Resource Allocation for V2X Communications. Sensors.

[29] Parvini M., Javan M.R., Mokari N., Abbasi B., Jorswieck E.A. (2023). AoI-aware resource allocation for platoon-based C-V2X networks via multi-agent multi-task reinforcement learning. IEEE Trans. Veh. Technol..

